# Grapes

**Published:** 1884-07

**Authors:** 


### GRAPES.

Dr. Hartsen of Cannes recommends grapes as a valuable diet in fever. Grapes contain a considerable amount of hydro-carbonaceous matter together with a certain quantity of potassium salts, a combination which does not irritate, but on the contrary soothes the stomach, and consequently is used with advantage, even in dyspepsia. While considering the carbo-hydrates contained in the grape, we must not neglect the organic acids, particularly tannic acid. Dr. Hartsen thinks the nourishing influence of these acids too much neglected. It is known that they are changed to carbonic acid in the blood and excreted as carbonates in the urine. When fresh grapes are not to be had raisins or diluted wines may be used.

Remedy for Cramp.—A writer in the "British Medical Journal" says : "The best remedy for cramp is a band of cork. Cut a clean cork into thin slices and sew close together upon a piece of ribbon or tape an inch wide. It can be tied around the affected part and worn at night.

Cure for Hiccough.—Sit erect and inflate the lungs fully. Then, retaining the breath, bend forward slowly until the chest meets the knees. After slowly rising again to the erect position slowly exhale the breath. Repeat this process a second time, and the nerves will be found to have received an access of energy that will enable them to perform their natural functions.

